# CausalTrail: Testing hypothesis using causal Bayesian networks

**DOI:** 10.12688/f1000research.7647.1

**Published:** 2015-12-30

**Authors:** Daniel Stöckel, Florian Schmidt, Patrick Trampert, Hans-Peter Lenhof

**Affiliations:** 1 Centre for Bioinformatics, Saarland University, Saarbrücken, 66123, Germany; 2 Cluster of Excellence 'Multimodal Computing and Interaction', Computer Science, Saarland University, Saarbrücken, 66123, Germany

**Keywords:** software, Bayesian networks, causality, interventions, counterfactuals, GUI, expectation-maximisation, do-calculus

## Abstract

**Summary** Causal Bayesian Networks are a special class of Bayesian networks in which the hierarchy directly encodes the causal relationships between the variables. This allows to compute the effect of interventions, which are external changes to the system, caused by e.g. gene knockouts or an administered drug. Whereas numerous packages for constructing causal Bayesian networks are available, hardly any program targeted at downstream analysis exists. In this paper we present CausalTrail, a tool for performing reasoning on causal Bayesian networks using the do-calculus. CausalTrail's features include multiple data import methods, a flexible query language for formulating hypotheses, as well as an intuitive graphical user interface. The program is able to account for missing data and thus can be readily applied in multi-omics settings where it is common that not all measurements are performed for all samples.

**Availability and Implementation** CausalTrail is implemented in C++ using the Boost and Qt5 libraries. It can be obtained from https://github.com/dstoeckel/causaltrail

## Introduction

An important task in molecular biology is the experimental validation of new hypotheses. This, however, can prove to be an expensive and time-consuming endeavour. Computational methods that allow to assess hypotheses *in-silico* can, consequently, decrease costs and increase productivity considerably. A popular class of methods for this purpose are graphical models. Graphical models are statistical models for which the dependencies between its variables can be interpreted as a graph structure. Bayesian networks (BNs), a special class of graphical models, are frequently used in bioinformatics as they allow to model dependencies between biological entities as a directed acyclic graph. In a BN, an arc from parent to child is often assumed to model a causal relationship. This, however, is not true in general. Often, multiple equivalent BNs for one probability distribution with differing topological order exist. Hence, they encode different “causal” relationships. Pearl *et al.* showed under which conditions the dependencies in a BN do, in fact, model real causal effects and described a formal framework for causal reasoning ^1^. This framework, known as *do-calculus*, allows to examine hypothesis on how external changes ( *interventions*) affect a system’s behaviour. Examples for interventions in a causal BN (CBN) are to add/remove edges or to set a node to a constant value. The do-calculus allows the modeling of the effects of mutations, gene knockouts, or *counter-factual* questions such as “Would the patient have recovered when administered drug B, knowing that he did not recover when administered drug A?”. The ability to answer questions like this is essential for the study of gene regulation or the evaluation of treatment regimens and, therefore, should be well supported by appropriate tools.

For inferring BNs and learning their parameters various packages such as bnlearn ^2^, BANJO ^3^, BNFinder2 ^4^, or SMILE ^5^ exist. SMILE additionally provides the *graphical user interface (GUI)* GeNIe. The pcalg R package ^6^ allows to infer the structure of CBNs. Murphy ^7^ compiled an extensive list of available software for working with graphical models. Although many of the listed tools are able to conduct *Bayesian inference*, we only found one commercial tool, *BayesiaLab* ^8^, supporting *causal reasoning* through interventions. None of the tools seem to support counterfactual queries. With CausalTrail we provide a software for conducting causal reasoning using the do-calculus with which we attempt to fill the apparent lack of free tools in this area. Given a predefined CBN structure, CausalTrail infers parameters using an expectation maximization (EM) procedure that can cope with missing data. This makes CausalTrail applicable to multi-omics datasets where some measurements may be missing or must be discarded due to quality issues. After parameter learning the user can pose, possibly counterfactual, queries containing causal interventions. For the implementation of CausalTrail we put special emphasis on the performance and reliability of the implemented methods. We additionally provide a simple, but flexible query language for formulating hypotheses as well as a user friendly GUI. CausalTrail is licensed under GPLv3 and can be obtained from https://github.com/dstoeckel/causaltrail.

## Methods

### Implementation

CausalTrail is written in C++ and uses the Boost and Qt5 libraries, as well as the Google Test framework for unit tests. CBN topologies are read from *simple interaction format (SIF)* and *trivial graph format (TGF)* files. Experimental data must be provided as a whitespace separated matrix.

As CausalTrail does not directly support continuous variables, continuous input data must be discretised using one of the provided discretisation methods. The *ceil, floor,* and *round* methods discretise the inputs to the nearest integers. In contrast thresholding-based methods like the *arithmetic* or *harmonic mean, median, z-score* and *fixed threshold* methods create binary output. The bracket medians and Pearson-Tukey ^9^ procedure create three or more output classes. Discretisation methods can be directly specified using the GUI or via a JSON-based input file.

For parameter learning the EM procedure described by Koller *et al.* ^10^ is used in order to account for missing values. To avoid local minima, the EM algorithm is restarted multiple times using different initialization schemes. For Bayesian reasoning, we implemented the *variable elimination* algorithm (cf. Koller *et al.* ^10^). Counterfactuals are computed using the twin network approach ^1^.

CausalTrail uses an intuitive query language for formulating hypotheses. Every query starts with a ’ ?’ followed by a list of nodes for which the posterior probability of a certain state. Alternatively it is possible detect the most likely state of a variable using the argmax function. It is possible to condition on a list of nodes using the ’ |’ character. Similarly, interventions can be stated after ’ !’. Possible interventions are: fixed value assignments ( N = v), edge additions between nodes N and M (+N M) and edge removals (-N M). Example queries are given in Table 1.

**Table 1.** Example queries for the Sachs *et al.* ^12^ dataset. High phosphorylation levels for ERK increase the likelihood of AKT being phosphorylated. In contrast, no such influence is detectable for PKA. The last two rows show the effect of *conditioning* on ERK.

Query	Result	Probability
? argmax(AKT) ? argmax(AKT) ! do ERK = 2 ? argmax(AKT) ! do ERK = 0	1 2 0	0.354 0.774 0.691
? argmax(PKA) ? argmax(PKA) ! do ERK = 2 ? argmax(PKA) ! do ERK = 0	2 2 2	0.336 0.336 0.336
? argmax(PKA) | ERK = 2 ? argmax(PKA) | ERK = 0	2 0	0.505 0.423

Multiple network instances can be loaded and used in the same session. The session itself can be saved and restored at any point in time. Network layouts are computed using a force-directed algorithm or, if installed, using Graphviz ^11^.

### Operation

We developed and tested CausalTrail under Ubuntu Linux 14.04. Compiling the code under Windows is possible using MSVC 2015, but not officially supported.

When invoking the command line application, a file containing the observations, a file specifying how the observed variables should be discretised, as well as the used network topology need to be specified. Once the input files are read, CausalTrail computes and prints the parameters of the Bayesian network. After the parameters have been computed, the user can enter queries in the query language.

**Figure 1.**
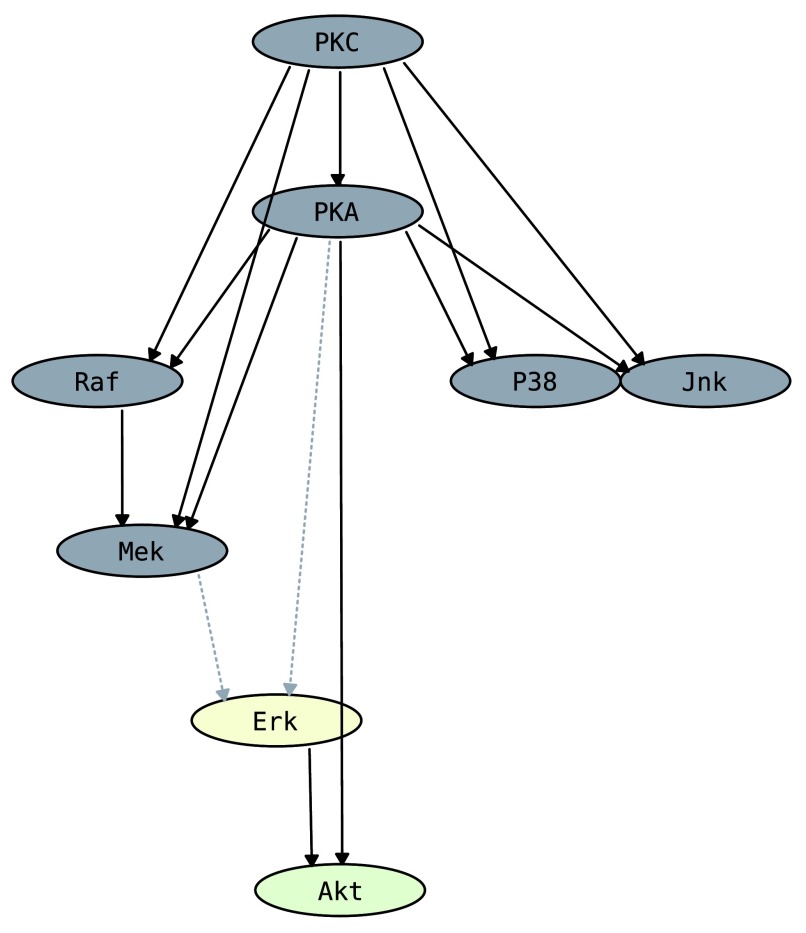
The CBN constructed by Sachs *et al.* ^12^, rendered using CausalTrail’s SVG export functionality. Nodes represent proteins and edges phosphorylation events. Nodes for which probabilities should be computed are coloured light green. Nodes with fixed values due to an intervention are coloured light yellow. The dashed edges are not considered during evaluation due to the intervention on ERK.

The graphical user interface workflow is similar to the CLI workflow and all functionality available in the CLI is also available in the GUI. First, the user needs to load a network topology, followed by observational data. Then, the discretisation methods to be used for the variables can be selected or loaded from a file. Queries can be entered manually via a text field or built interactively by right-clicking on the network nodes and edges. In the first case, queries are automatically checked for validity while typing. The nodes and edges involved in a query are highlighted. Counterfactual queries can be generated by conditioning and creating an intervention on a variable simultaneously.

## Use-case

We demonstrate an application of CausalTrail, using the protein signaling network inferred by Sachs *et al.* ^12^ (see Figure 1). The authors validated the existence of the arc between ERK and AKT by showing that an intervention on ERK has an effect on AKT, but no effect on PKA. To this end, the phosphorylation of AKT and PKA was measured with ERK being (i) unperturbed, (ii) stimulated, and (iii) knocked down using siRNAs. Whereas the stimulation of ERK had no effect on PKA, it lead to an increase in AKT phosphorylation. For the knockdown, again no change of PKA phosphorylation could be detected whilst the phosphorylation of AKT dropped slightly below the level of the unperturbed case. To test whether the inferred network models the experimental data faithfully, we used the dataset and topology provided by Sachs *et al.* ^12^ to train the parameters of a CBN and examined the arc between ERK and AKT more closely. To this end, we discretised each protein’s phosphorylation level into the classes low (0), medium (1), and high (2) using the *bracket medians* procedure. We then computed the most likely phosphorylation state of AKT and PKA in (i) unperturbed, (ii) stimulated, and (iii) ERK knockout cells, which we modelled using interventions that fix the ERK phosphorylation level to high and low respectively. The computed queries are given in Table 1. We find that the stimulation of ERK leads to an increased AKT phosphorylation level. When ERK is knocked out AKT phosphorylation drops to low showing that the previous increase was, in fact, mediated by ERK. In contrast the activity of ERK has no effect on the phosphorylation of PKA. Note that using an intervention is essential for this observation as conditioning on ERK would render PKA dependent on ERK resulting in a different prediction (see bottom lines in Table 1).

## Discussion

CausalTrail enables its users to harness the additional expressivity offered by the do-calculus to formulate and test biological hypotheses *in-silico*. In addition to basic interventions, CausalTrail supports the evaluation of counterfactuals using the twin network approach. To the best of our knowledge, it is the only available tool that offers this functionality. Our software offers efficient implementations for parameter learning and query evaluation that allow examining experimental data in an interactive fashion. The showcased application of causal reasoning demonstrates that CausalTrail may be a valuable addition to a bioinformatician’s toolbox for the interpretation of Bayesian networks.

## Software availability

1. URL link to the author’s version control system repository containing the source code ( https://github.com/dstoeckel/causaltrail)
2. Link to source code at time of publication ( https://github.com/F1000Research/causaltrail)
3. Link to archived source code at time of publication ( http://dx.doi.org/10.5281/zenodo.35611)
4. Software license (GNU General Public License version 3)
