# Supplementary material for: CausalTrail: Testing hypothesis using causal Bayesian networks
Source: F1000Res. 2015 Dec 30;4:ISCB Comm J-1520. [Version 1] doi: 10.12688/f1000research.7647.1 (PMC4743151; doi:10.12688/f1000research.7647.1)
Supplement: Peer review history

# Peer review: Referee response for version 1

**DOI:** 10.5256/f1000research.8234.r11738

**Published:** 2016-01-29

**Authors:** Karen Sachs

**Affiliations:** 1 School of Medicine, Stanford University, Stanford, CA, USA

The authors fill a void in the computational biology toolkit, as I am not aware of a software package for performing inference on nodes, only packages for structure learning. This important functionality is a useful contribution.

My main critique is on the usability - if this is meant to target biologists, it would be helpful to make the file formats very simple, in particular most biologists use Excel for their data, so it would be helpful if they could specify the model and the data in a csv or txt file.

I have read this submission. I believe that I have an appropriate level of expertise to confirm that it is of an acceptable scientific standard.

# Peer review: Referee response for version 1

**DOI:** 10.5256/f1000research.8234.r11879

**Published:** 2016-01-18

**Authors:** Maxime Gasse

**Affiliations:** 1 DM2L - Data Mining and Machine Learning, LIRIS - Computer Science Laboratory for Image Processing and Information Systems, UMR CNRS 5205, University of Lyon, Lyon, France

An interesting tool that provides an easy-to-use user interface to play with causal Bayesian networks. Do-calculus provides a safe and sound framework for reasoning with effects, causes and interventions. It is good see the development of such kind of software to help in the adoption of CBNs and do-calculus to express and compare the results of experimental studies.

I can not judge on the protein signaling use-case presented in the article, however the methods employed to perform inference and parameter learning are standard and appropriate. Still, more details about the parameter learning implementation would be welcome: is there any kind of regularization done along with EM? Is it possible to specify some priors?

The tool is young and suffers from occasional crashes (I encountered some while testing), however the code is open source which should greatly help in fixing bugs or implementing new functionalities upon it. Some ideas of such functionalities which could improve the software:

- being able to specify toy Bayesian network structures by hand using the GUI
- being able to specify and modify the CPTs by hand in the GUI
- being able to generate data from the network, from the joint distribution p(x), conditional distributions p(y|x), post-intervention distributions p(y|do(x)) or even mixing both p(y|y,do(z))

I have read this submission. I believe that I have an appropriate level of expertise to confirm that it is of an acceptable scientific standard.
